# Bibliometric and visualized analysis of metal-organic frameworks in biomedical application

**DOI:** 10.3389/fbioe.2023.1190654

**Published:** 2023-05-10

**Authors:** Sanyang Yu, Kaihao Xu, Zhenhua Wang, Zhichang Zhang, Zhongti Zhang

**Affiliations:** ^1^ The VIP Department, School and Hospital of Stomatology, China Medical University, Shenyang, China; ^2^ Department of Physiology, School of Life Sciences, China Medical University, Shenyang, China; ^3^ Department of Computer, School of Intelligent Medicine, China Medical University, Shenyang, China

**Keywords:** metal-organic frameworks (MOFs), biomedical applications, biomaterial, bibliometrics, data visualization

## Abstract

**Background:** Metal-organic frameworks (MOFs) are hybrid materials composed of metal ions or clusters and organic ligands that spontaneously assemble via coordination bonds to create intramolecular pores, which have recently been widely used in biomedicine due to their porosity, structural, and functional diversity. They are used in biomedical applications, including biosensing, drug delivery, bioimaging, and antimicrobial activities. Our study aims to provide scholars with a comprehensive overview of the research situations, trends, and hotspots in biomedical applications of MOFs through a bibliometric analysis of publications from 2002 to 2022.

**Methods:** On 19 January 2023, the Web of Science Core Collection was searched to review and analyze MOFs applications in the biomedical field. A total of 3,408 studies published between 2002 and 2022 were retrieved and examined, with information such as publication year, country/region, institution, author, journal, references, and keywords. Research hotspots were extracted and analyzed using the Bibliometrix R-package, VOSviewer, and CiteSpace.

**Results:** We showed that researchers from 72 countries published articles on MOFs in biomedical applications, with China producing the most publications. The Chinese Academy of Science was the most prolific contributor to these publications among 2,209 institutions that made contributions. Reference co-citation analysis classifies references into 8 clusters: synergistic cancer therapy, efficient photodynamic therapy, metal-organic framework encapsulation, selective fluorescence, luminescent probes, drug delivery, enhanced photodynamic therapy, and metal-organic framework-based nanozymes. Keyword co-occurrence analysis divided keywords into 6 clusters: biosensors, photodynamic therapy, drug delivery, cancer therapy and bioimaging, nanoparticles, and antibacterial applications. Research frontier keywords were represented by chemodynamic therapy (2020–2022) and hydrogen peroxide (2020–2022).

**Conclusion:** Using bibliometric methods and manual review, this review provides a systematic overview of research on MOFs in biomedical applications, filling an existing gap. The burst keyword analysis revealed that chemodynamic therapy and hydrogen peroxide are the prominent research frontiers and hot spots. MOFs can catalyze Fenton or Fenton-like reactions to generate hydroxyl radicals, making them promising materials for chemodynamic therapy. MOF-based biosensors can detect hydrogen peroxide in various biological samples for diagnosing diseases. MOFs have a wide range of research prospects for biomedical applications.

## 1 Introduction

Metal-organic frameworks (MOFs) are hybrid materials composed of metal ions or clusters and organic ligands that spontaneously assemble via coordination bonds to create intramolecular pores (James, 2003; Kitagawa, 2014). They are a class of crystalline materials with ultra-high porosity and substantial internal surface area (Zhou et al., 2012). MOFs can be synthesized using metal ions or metal-containing clusters and organic ligands via microwave-assisted (Chen et al., 2019), sonochemical (Yu K. et al., 2021), solvothermal (Wang et al., 2019), mechanochemical (Głowniak et al., 2021), microfluidic (Hu C. et al., 2020), dry-gel conversion (Zhuang et al., 2020). The structural parameters of MOFs and their derived functional materials can be tuned by adjusting the metal nodes and ligands in MOFs and controlling their morphology (Liu et al., 2021). Due to their versatility and ability to be tailored for specific applications, MOFs have been used in an extensive range of adsorption (Zhang et al., 2022b), gas storage and separation (Li et al., 2019), catalysts (Rosen et al., 2022), sensors (Yuan et al., 2022). The application of MOFs in the biomedical field has become a rapidly growing research hotspot (Gupta et al., 2016). Due to their stability, biocompatibility, biodegradability, and low cytotoxic effects, MOFs are a promising biomaterial for many biomedical applications (Awasthi et al., 2022). MOFs and their derivatives have been studied for biosensing (Zhang et al., 2022a; Yan et al., 2023; Yu et al., 2023), drug delivery (Abánades Lázaro and Forgan, 2019), bioimaging (Wang, 2017), and antimicrobial activities (Yang and Yang, 2020; Pettinari et al., 2021).

Bibliometrics is a field of study that measures and analyzes the influence of scholarly publications using quantitative approaches. It provides a quantifiable measurement for the significance of a research study, typically by measuring the number of citations a publication receives (Cooper, 2015). Using bibliometric analysis, it is possible to assess the impact of individual researchers, institutions, journals, countries, and research topics. Bibliometrics can also help researchers identify research trends, recognize essential research questions, and evaluate the impact of individual research projects. Bibliometrics has become an essential instrument for evaluating research and research institutes.

Many articles have reviewed the biomedical applications of MOFs; however, the field needs bibliometric analysis. To fill this gap, we performed a systematic and comprehensive bibliometric analysis to overview the current research trends concerning the biomedical applications of MOFs. Our study offers a bibliometric analysis of the biomedical applications of MOFs in terms of the annual growth of publications, countries/institutions/authors, most influential journals, references, and keywords to provide a holistic view. Additionally, keyword co-occurrence networks analysis and burst keyword detection based on VOSviewer and CiteSpace were utilized to describe and discuss research classifications and hot topics trends for MOFs biomedical applications.

## 2 Methods

### 2.1 Systemic search strategy

Searched in the Web of Science Core Collection were conducted using the following search phrases: TS=(“Metal-Organic Framework” OR “Metal Organic Framework”) AND TS= (“antibacteri*” or “antimicrob*” or “bioassay*” or “biodevice*” or"bioelectronic*” or “bioimaging” or biomarker or “biomedic*” or “biosens*” or “cancer” or “diagnos*” or “disease” or “drug deliver*” or “electronic skin” or “healthcare” or “human breath monitoring” or “health monitor*” or “immunosens*” or “medic*” or “medical device” or “nanomedic*” or “stem cell engineer*” or "*therap*” or “tumor” or “theranos*” or ‘‘tissue engineering” or “wearable device*”) NOT AK=(“adsorption”) NOT TS=(“pesticides” or biofuel or ”*water treatment” or food pack* or food analysis or “environment* applicat*”) (Zhu et al., 2022; Gu et al., 2023). The retrieved publications were downloaded and managed in plain text file format, and record content includes full records and cited references. Due to the rapid rate of scientific research being published and updated, data retrieval and download was completed in one day on 19 January 2023.

### 2.2 Eligibility criteria

**FIGURE 1:**
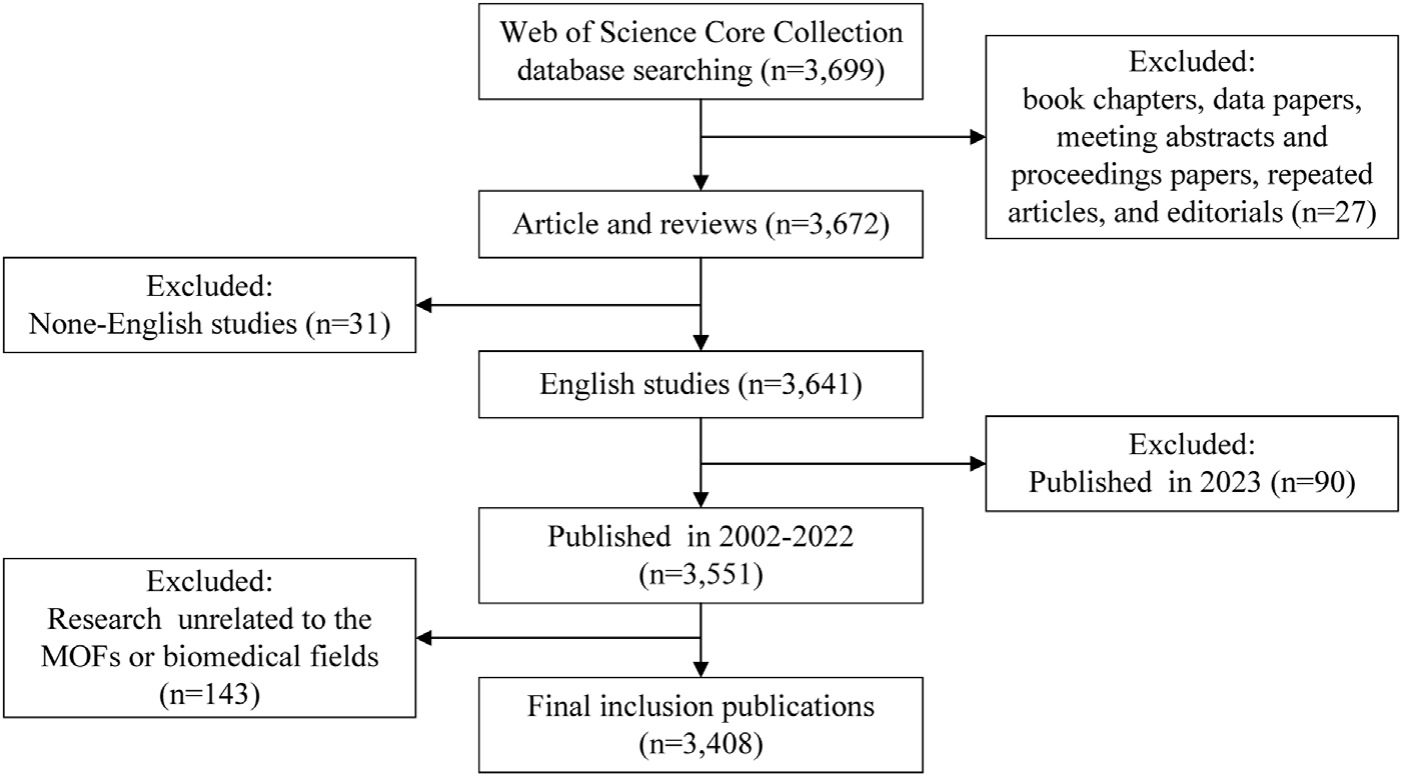
Criteria and flowchart for inclusion and exclusion of studies.

The inclusion criteria for the database were limited to studies that met the following conditions: 1) article and review article indexed in the database, 2) published and had sufficient data for analysis, 3) written in English, 4) published before the end of 2022, and 5) relevant to the research content. Any studies that did not meet these criteria were excluded from the analysis. In total, 291 studies were excluded. Figure 1 depicts the detailed process of publications inclusion and exclusion.

### 2.3 Bibliometric data analysis

Various characteristics of the papers were systematically described, including country or region, institution, author, journal, reference, and keywords. The downloaded and inspected data were integrated into the bibliometric analysis software to construct bibliometric maps.

RStudio V4.2.2 with the Bibliometrix package installed was used to extract and analyze the main message of downloaded publications (Aria and Cuccurullo, 2017). The number of publications and annual growth condition were extracted in the overview section; the information on journals was extracted in the sources section; the citation of publications, reference citations, and keywords occurrence frequency was extracted in the document section. All the extracted data were saved in CSV format.

VOSviewer 1.6.18 was used to analyze countries/regions, organizations, and keywords, and the counting method was set to full counting (Van Eck and Waltman, 2010). For analyzing the number of publications and citation frequency of countries and organizations, the type of authorship was selected as co-authorship or citation, and the unit of analysis is set as countries and organizations respectively. For constructing the keywords occurrence network, the type of authorship was selected as co-occurrence, and the unit of analysis was selected as author keywords.

CiteSpace 6.1R6 was used to conduct journal biplot overlay analysis, references co-citation analysis, and burst keyword identification (Chen, 2018). With 2 years as a slice, the subsequent analysis was carried out. The node types were selected as keyword and reference for clustering analysis, and the burst detection function was used to detect burst keywords. JCR Journal Maps was selected under the overlay map option, the Z scores method was selected, and a journal biplot overlay analysis was performed.

## 3 Results

A total of 3,408 publications in the Web of Science Core Collection met the criteria to be included in the study.

### 3.1 Publications and annual growth

**FIGURE 2:**
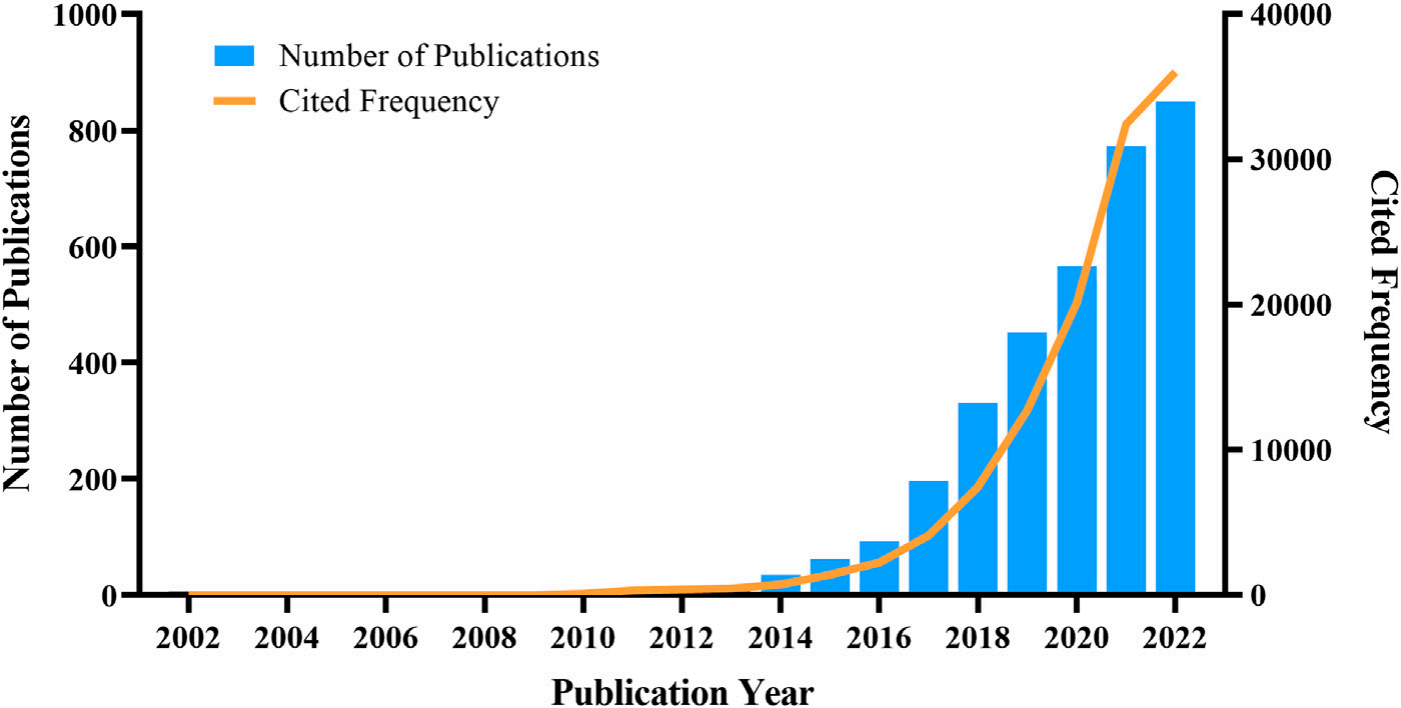
Trends in the number of publications from 2002 to 2022.

We analyzed the yearly growth of the study based on the publication year of the articles. The number of published studies increased annually between 2002 and 2022 (Figure 2), with an annual growth rate of 40.11%, indicating that biomedical applications of MOFs are attracting increasing attention from researchers. Prior to 2014, researchers paid little attention to MOFs in biomedical research. However, there has been a rapid rise in related studies since then.

### 3.2 Countries/institutions/authors

The included publications come from 14,806 authors from 2,209 institutions in 72 countries.

**FIGURE 3:**
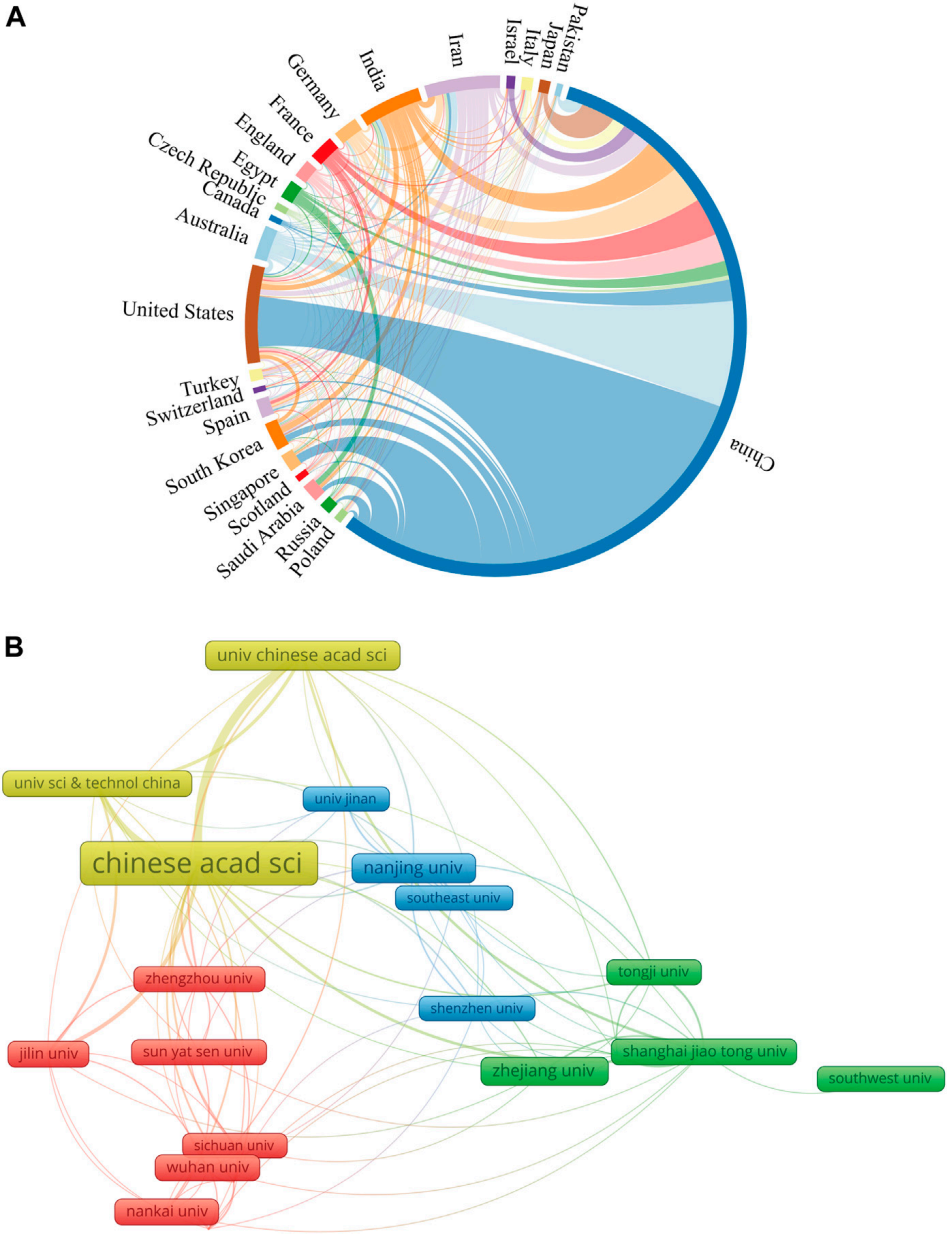
The cooperation network map of countries/regions and institutions. **(A)** Countries/regions; **(B)** Institutions.

The top ten countries in terms of the number of publications are shown in Table 1. Researchers from China published the highest number of papers (*n* = 2,466, 72.36%), followed by the United States (*n* = 283, 8.30%), with publications from China accounting for more than half of the total. The country with the highest number of publication citations was China (*n* = 84,650/Ave = 34.33), followed by the United States (19,668/69.50) and South Korea (7,234/88.22). Researchers from France published studies that received the most citations on average for each publication (90.43), followed by South Korea, Germany (75.48), the United States, and Australia (65.68). H-index is an indicator that is composed of multiple factors. It can be used to measure academic achievement (Hirsch, 2007). China ranked first on the H-index (124), followed by the United States (69). We conducted a visual analysis of the collaboration among countries and constructed a collaboration network (Figure 3A). In this diagram, the number of countries published is represented by the length of the arc, and the intensity of cooperation between countries is depicted by the width of the lines that link the arcs. China showed close collaborations with the United States, Australia, and Singapore, and the United States had active collaborations with Australia, India, and South Korea.

**TABLE 1:** Top 10 countries/regions and institutions.

Rank	Countries/regions	Count	H-index	Institutions	Count	H-index
1	China	2,466	124	Chinese Academy of Sciences	310	68
2	United States	283	69	Nanjing University	106	36
3	Iran	217	37	University of Chinese Academy of Sciences	101	39
4	India	180	35	Zhejiang University	95	32
5	Australia	97	34	Changchun Institute of applied Chemistry	80	41
6	South Korea	82	31	Jilin University	68	29
7	France	72	33	University of Science Technology of China	67	32
8	Germany	71	32	Wuhan University	67	27
9	Spain	54	26	Shanghai Jiao Tong University	66	24
10	Egypt	54	17	Centre National de la Recherche Scientifique	63	30

The top ten institutions based on the number of included publications were summarized in Table 1. The most productive institution was the Chinese Academy of Sciences (310 publications/15,700 citations), followed by Nanjing University (106/6,022) and the University of Chinese Academy of Sciences (101/5,307). Figure 3B shows the collaborative network of the institutions. The number of articles issued is indicated by the label size, and the closeness of cooperation is shown by the line thickness. The Chinese Academy of Sciences collaborated most closely with the University of Chinese Academy of Sciences and the University of Science and Technology of China.

The average number of co-authors per document was 6.63, and only 22 publications had a single author, revealing a significant amount of collaboration amongst the researchers working in biomedical MOFs.

### 3.3 Journals

**FIGURE 4:**
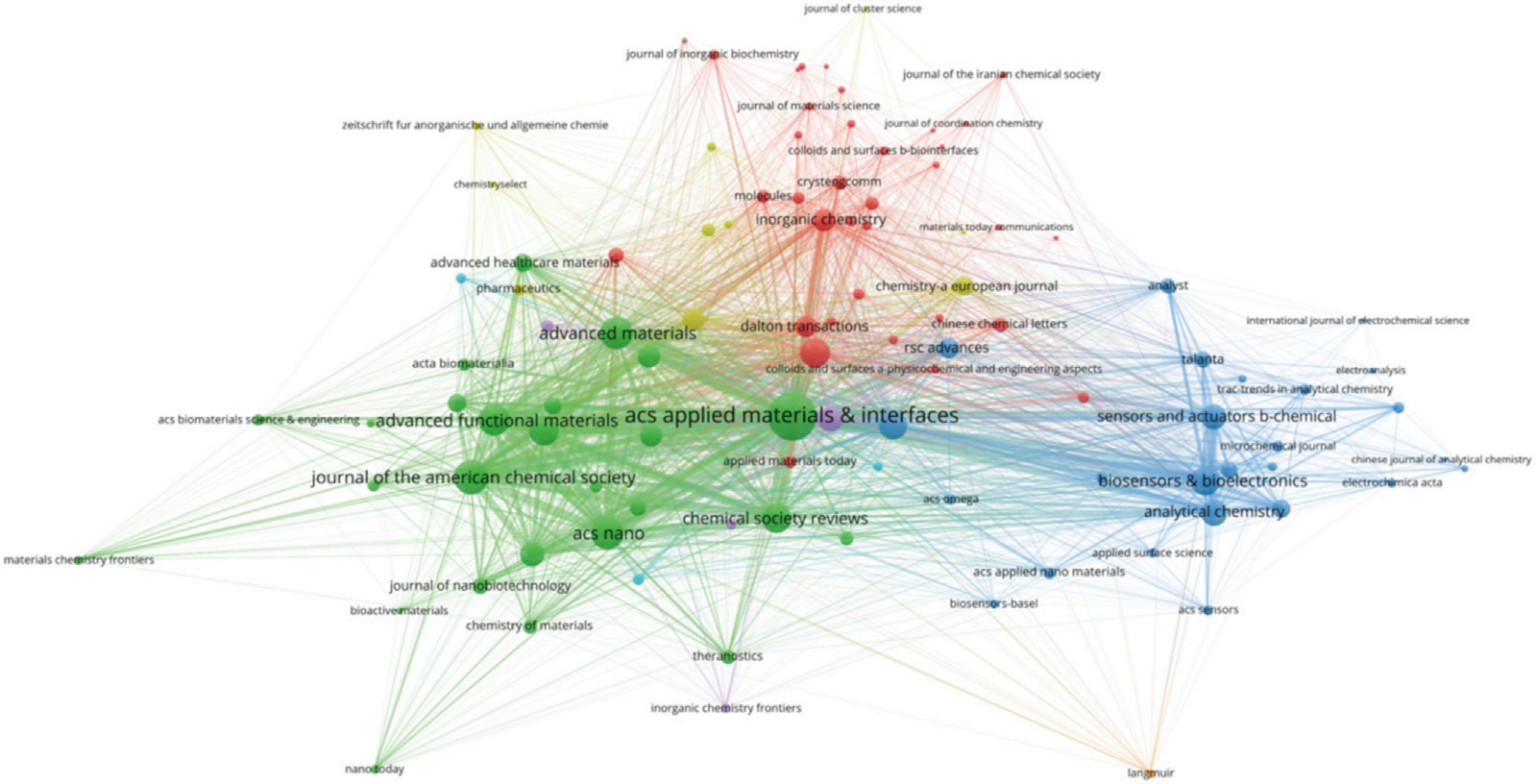
The collaboration network map of journeys.

A total of 440 journals published research on biomedical applications of MOFs. We listed core journals in MOFs in biomedical applications according to Bradford’s Law (Heine, 1998) (Table 2). The most relevant journal was ACS Applied Materials Interfaces (291 publications/9,385 citations), followed by Sensors and Actuators B Chemical (100/3,195) and Chinese Chemical Letters (99/2,905). The journals obtained from the study are mainly related to chemistry, sensors, nanoscience, or biomaterials, indicating that MOFs have promising research prospects in these fields. Citation relationships between scientific journals indicate the exchange of knowledge, with the citing studies representing the frontiers of knowledge and the referenced studies forming the foundation (Xu et al., 2022). The collaborative network between related journals as shown in Figure 4, with the size of the circles indicating the centrality of the nodes. ACS Applied Materials & Interfaces, Journal of the American Chemical Society, Advanced Materials, Lacs Nano, and Advanced Functional Materials were the most popular journals for publishing research on this topic.

**TABLE 2:** Core journals and top 10 co-cited references.

Rank	Core journals titles	Count	H-index	Co-cited references titles	DOI	Count
1	ACS applied materials interfaces	219	58	Porous metal–organic-framework nanoscale carriers as a potential platform for drug delivery and imaging (Horcajada et al., 2010)	10.1038/NMAT2608	418
2	Sensors and actuators B chemical	100	34	The Chemistry and Applications of Metal-Organic Frameworks (Furukawa et al., 2013)	10.1126/SCIENCE.1230444	357
3	Chinese chemical letters	99	26	Metal-Organic Frameworks in Biomedical field (Horcajada et al., 2012)	10.1021/CR200256V	281
4	Biosensors bioelectronics	97	39	Metal–Organic Framework (MOF)-Based Drug/Cargo Delivery and Cancer Therapy (Wu and Yang, 2017)	10.1002/ADMA.201606134	266
5	Analytical chemistry	92	36	One-pot Synthesis of Metal−Organic Frameworks with Encapsulated Target Molecules and Their Applications for Controlled Drug Delivery (Zheng et al., 2016)	10.1021/JACS.5B11720	206
6	Journal of materials chemistry B	78	27	Metal-Organic Framework Materials as Chemical Sensors (Kreno et al., 2012)	10.1021/CR200324T	199
7	Microchimica acta	72	16	Size-Controlled Synthesis of Porphyrinic Metal−Organic Framework and Functionalization for Targeted Photodynamic Therapy (Park et al., 2016)	10.1021/JACS.6B00007	196
8	Advanced functional materials	68	34	Nanoscale Metal–Organic Frameworks for Biomedical Imaging and Drug Delivery (Della Rocca et al., 2011)	10.1021/AR200028A	192
9	Chemical engineering journal	61	21	Nanoscale Metal−Organic Framework for Highly Effective Photodynamic Therapy of Resistant Head and Neck Cancer (Lu et al., 2014a)	10.1021/JA508679H	180
10	Analytica chimica acta	56	18	Introduction to Metal−Organic Frameworks (Zhou et al., 2012)	10.1021/CR300014X	170
11	Dalton transactions	56	20	—	—	—
12	Inoraganic chemistry	54	27	—	—	—
13	Talanta	53	24	—	—	—
14	Chemical communications	52	28	—	—	—

**FIGURE 5:**
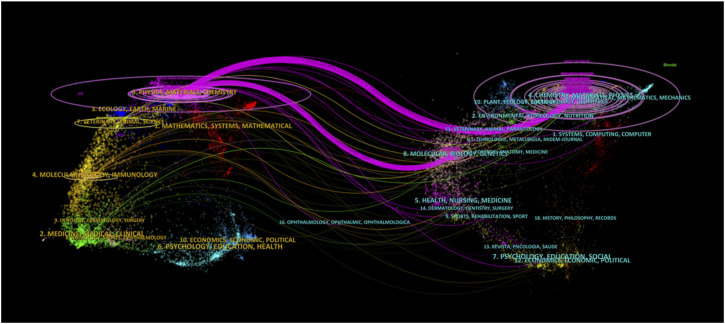
The dual-map overlays of journeys.

Dual-map overlay is a method designed by Chaomei Chen and Loet Leydesdorff for assessing, comparing, and contrasting publication portfolio features (Chen and Leydesdorff, 2014). A dual-map overlay was used to illustrate the citation linkages between journals (Figure 5). The left side of the figure shows the citing journals, and the right side shows the cited journals, with colored lines in the middle representing the connections between them. The analysis revealed that articles published in Physics/Materials/Chemistry journals typically cite articles from journals related to Chemistry/Materials/Physics and Molecular/Biology/Genetics.

### 3.4 References

A total of 130,835 references appeared in the included studies, the top ten references cited across the included studies as shown in Table 2. Porous metal-organic-framework nanoscale carriers as a potential platform for drug delivery and imaging was the most frequently cited reference (Horcajada et al., 2010).

**FIGURE 6:**
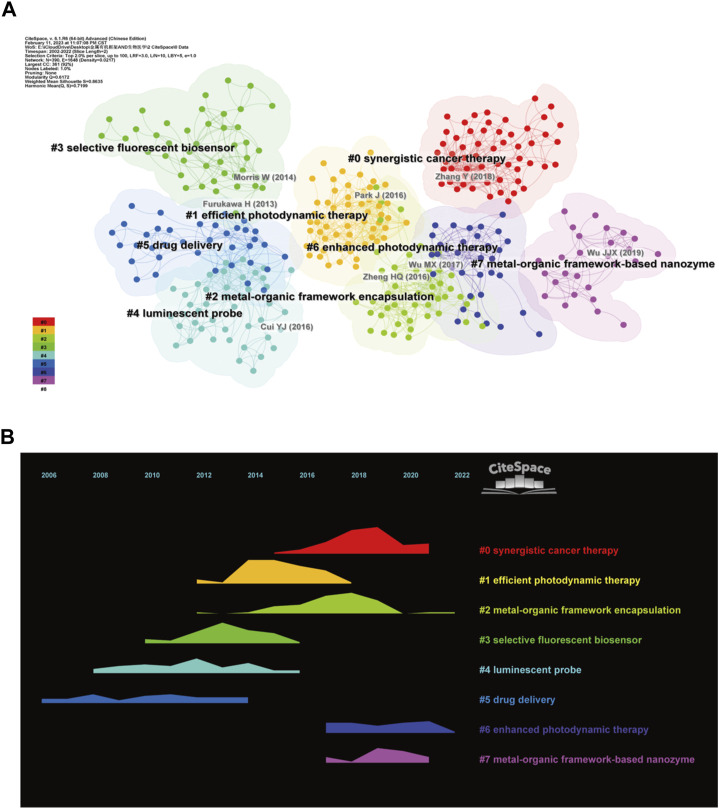
Reference co-citation map. **(A)** Reference clustering map; **(B)** Reference clustering temporal evolution.

The network of the reference co-citation was conducted to reveal the knowledge base (Figure 6A). Using 2 years as a slice, the top 2% of the articles were taken for cluster analysis. Upon completing the clustering process, the modularity Q score and weighted mean silhouette S were higher than 0.5, with values of 0.6172 and 0.8755, respectively. These two parameters indicate that the network was suitably divided into clusters with low inter-cluster connectivity and satisfactory homogeneity within clusters. The clustering markers for the study were the index terms extracted from the literature. The largest cluster was numbered #0 marked as “synergistic cancer therapy” (Li et al., 2017; Lan et al., 2018; Zhang et al., 2018), then in decreasing size were cluster #1 “efficient photodynamic therapy” (Lu K. et al., 2014; Park et al., 2016; Lismont et al., 2017), cluster #2 “metal-organic framework encapsulation” (Liang et al., 2015; Zheng et al., 2016; Lian et al., 2017), cluster #3 “selective fluorescent” (Morris et al., 2014; Zhang et al., 2014; Rodenas et al., 2015), cluster #4 “luminescent probe” (Kreno et al., 2012; Hu et al., 2014; Li et al., 2016), cluster #5 “drug delivery” (Cunha et al., 2013; Furukawa et al., 2013; Zhuang et al., 2014), cluster #6 “enhanced photodynamic therapy” (Wu and Yang, 2017; Lu et al., 2018; Simon-Yarza et al., 2018), cluster #7 “metal-organic framework-based nanozyme” (Huang et al., 2019; Liu et al., 2019; Wu et al., 2019). The temporal evolution of the reference co-citation analysis (Figure 6B) shows that cluster #5 “drug delivery” was one of the first clusters to emerge, and cluster #6 “enhanced photodynamic therapy” maintained a high intensity between 2020 and 2022. The results show that the application of MOFs in the biomedical field started with drug delivery. A new research trend in recent years is enhanced photodynamic therapy.

### 3.5 Keywords

**FIGURE 7:**
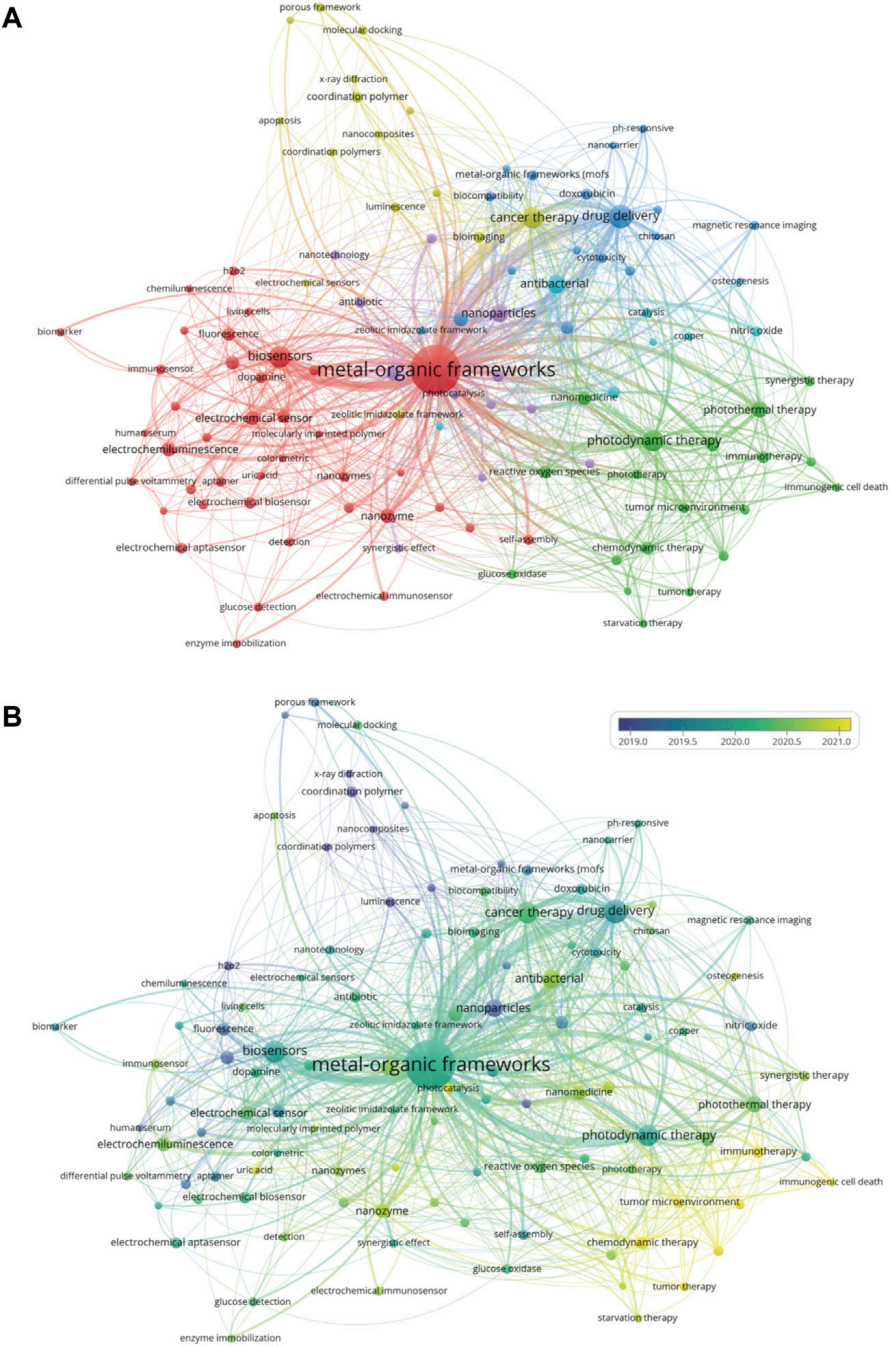
Visualized analysis of keyword co-occurrence. **(A)** Keywords clustering map; **(B)** Keywords clustering temporal evolution.

Investigating the author keywords of all publications on a topic can reveal the research direction of the field as they generally represent the main content of a paper. To further identify the main research directions of MOFs in biomedical fields, we analyzed the author keywords in each article in the database using VOSviewer. After merging synonyms, Figure 7A depicts a co-occurrence network of author keywords that appear more than ten times. In this diagram, the size of the label represents the occurrence frequency of the keyword, while the line thickness represents the connection strength. A total of 110 keywords were found, and these keywords were organized into 6 different clusters. The largest cluster is red, representing biosensors; the second largest cluster is green, representing photodynamic therapy; the third cluster is blue, representing drug delivery; the fourth cluster is yellow, representing cancer therapy and bioimaging; the fifth cluster is purple, representing nanoparticles; and the sixth cluster is sky blue, representing antibacterial applications. Table 3 summarizes the 20 most frequently occurring author keywords, suggesting that MOFs have gained importance in research regarding biosensing, cancer therapy, photodynamic therapy, drug delivery, and bioimaging. Figure 7B shows the temporal evolution of author keywords that appear more frequently than ten times, from blue to yellow coloration indicating increasingly new keywords. The keyword of osteogenesis, representing tissue engineering studies, appears around 2021, suggesting that the potential prospect of MOFs in tissue engineering applications is gaining increased attention (Li M. et al., 2022). In addition, immunogenic cell death, tumor microenvironment, and other related words also likely represent newly emerging hotspots.

**TABLE 3:** Top 20 author keywords.

Rank	Keywords	Count	Total link strength	Rank	Keywords	Count	Total link strength
1	Metal-organic frameworks	1,195	1,290	11	ZIF-8	47	68
2	Drug delivery	171	297	12	Nanomaterials	46	75
3	Biosensors	142	179	13	Electrochemiluminescence	46	51
4	Cancer therapy	134	262	14	Chemotherapy	45	96
5	Photodynamic therapy	130	237	15	Nanomedicine	40	76
6	Antibacterial	95	125	16	Glucose	40	54
7	Nanoparticles	79	133	17	Chemodynamic therapy	37	71
8	Photothermal therapy	66	124	18	Fluorescence	37	52
9	Electrochemical sensor	50	67	19	Reactive oxygen species	34	69
10	Nanozyme	48	70	20	Hydrogen peroxide	34	53

**FIGURE 8:**
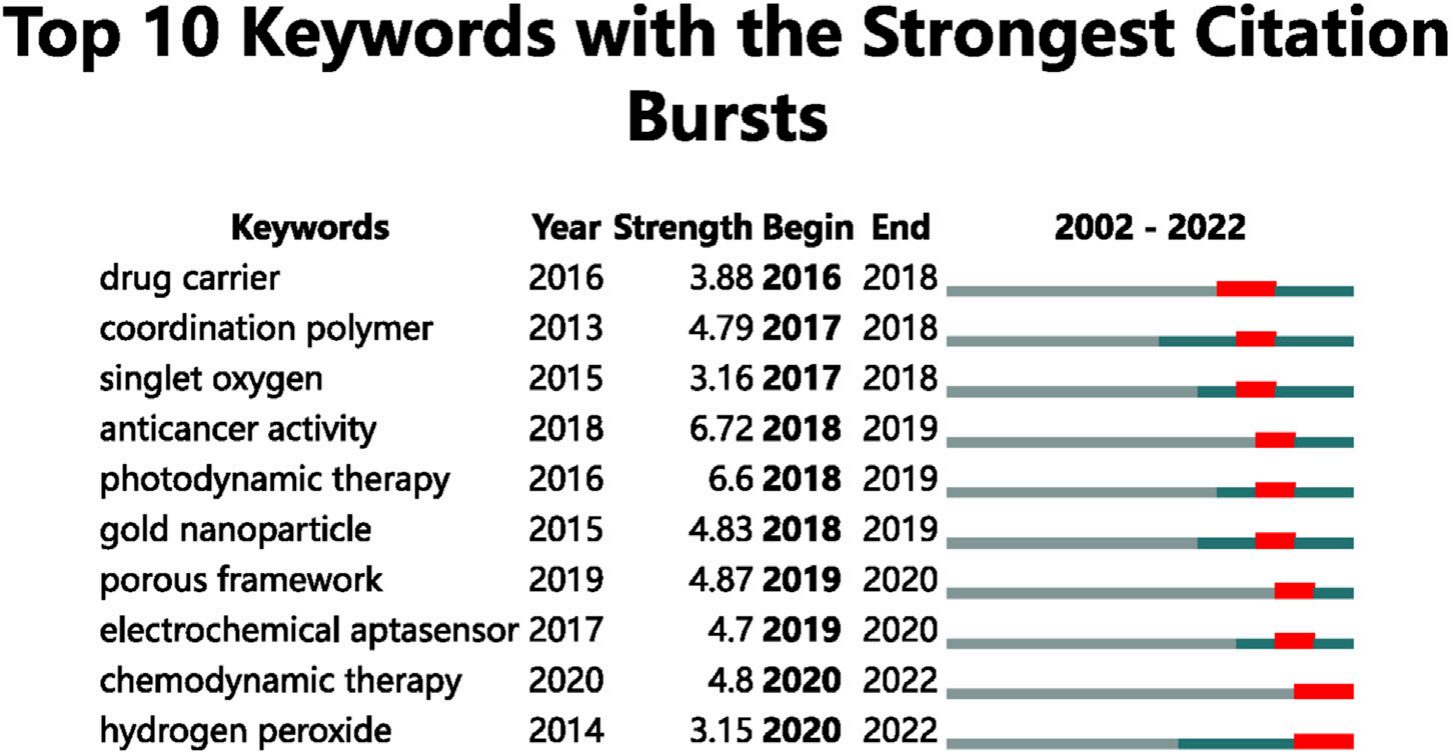
Top 10 keywords with the strongest citation bursts.

Based on the top ten keywords with the most prominent citation bursts, an analysis of the trend of hotspots was conducted (Figure 8). The burst keywords for 2016 to 2018 were drug carrier (2016–2018), coordination polymer (2017-2018), and singlet oxygen (2017-2018). The burst keywords for 2018 to 2019 were anticancer activity (2018-2019), photodynamic therapy (2018-2019), and gold nanoparticle (2018-2019). The burst keywords for 2019 to 2020 were porous framework (2019-2020) and electrochemical aptasensor (2019-2020). The burst keywords for 2020 to 2022 were chemodynamic therapy (2020–2022) and hydrogen peroxide (2020–2022).

## 4 Discussion

### 4.1 General information

In total, 3,408 SCI papers published between 2002 and 2022 in the field of biomedical applications involving MOFs were included in this study. China produced the most publications (2,466/72.36%), and the United States came in second (283/8.3%). The institution with the most publications was the China Academy of Sciences. ACS Applied Materials Interfaces was the most published journal. Porous metal-organic-framework nanoscale carriers as a potential platform for drug delivery and imaging (Horcajada et al., 2010) was the most frequently cited reference.

### 4.2 Research direction

The network visualization of the co-occurrence author keywords was constructed to identify the main research areas in which MOFs are used in biomedical applications (Figure 7A). The research areas were categorized in the following directions: biosensors, cancer therapy and photodynamic therapy, drug delivery, bioimaging, nanoparticles, and antibacterial applications.

In biosensor research, MOFs have been utilized as sensitive element carriers, enzyme mimics, electrochemical signaling, optical signaling, and gas sensing (Du L. et al., 2021). Afzalinia et al. used fluorescence resonance energy transfer and “sandwich” hybridization of oligonucleotides to make a biosensor. The fluorophore was a La(III)-MOF and the quencher was Ag nanoparticles, modified with appropriate aptamer chains for the intended function. This biosensor was optimized for miRNA-155 detection in clinical applications (Afzalinia and Mirzaee, 2020). Hüseyin Kıyıkçı et al. developed a straightforward amperometric biosensor to detect sialic acid, which uses Co/2Fe MOF (an oxidase-mimicking). It was successfully used to detect free silicic acid after the release of attached silicic acid molecules in A549, GD3, and HeLa cell lines (Kıyıkçı et al., 2023).

In cancer therapy including photodynamic therapy, MOF-based nanoplatforms can be utilized to treat cancer effectively using several single and combination therapies, including radiotherapy, chemotherapy, chemodynamic therapy, phototherapy (photothermal therapy and photodynamic therapy), starvation therapy, and immunotherapy (Yang et al., 2023). Among them, in photodynamic therapy, MOFs have the potential to act as carriers for loading photosensitizers or to increase the number of photosensitizers in target cells. They are capable of constructing multifunctional systems which help to refine the tumor microenvironment, therefore enhancing the efficacy of photodynamic therapy when used in combination therapy (Ye et al., 2022). Du et al. developed D-arginine-loaded MOF nanoparticles for improving the radiosensitivity of osteosarcoma (Du C. et al., 2021). Ding et al. created an innovative multifunctional platform based on the core-shell structure of 5-aminolevulinic acid@UiO-66-NH-FAM@zirconium-pemetrexed. It exhibited excellent folate targeting capacity and highly effective anticancer efficacy by combining chemotherapy with photodynamic therapy (Ding et al., 2022). Tian et al. developed a MOF-199 nanoplatform with vitamin k3 for enhanced chemodynamic therapy. It efficiently accumulates in tumor tissues due to the enhanced permeability and retention effect (Tian et al., 2021). Lan et al. created a new titanium MOF for hypoxia-tolerant type I photodynamic therapy. The structure called Ti-TBP is made up of photosensitizing 5,10,15,20-tetra porphyrin ligands coordinated on secondary Ti-oxo chain building units. (Lan et al., 2019). Yu et al. designed and synthesized a new nanomedicine, CHC/GOx@ZIF-8, which is capable of dual-depriving lactate and glucose, to enhance the anti-tumor efficacy of the cancer starvation therapy (Yu J. et al., 2021). Shao et al. designed core–shell upconversion nanoparticle@porphyrinic MOFs for combinational therapy against hypoxic tumors. The nanoplatform for combining NIR light-triggered PDT and hypoxia-activated chemotherapy with immunotherapy to combat the current limitations of tumor treatment (Shao et al., 2020).

In drug delivery, it is possible to alter synthetic methods to produce MOFs in a nanoscale dimension or adjust MOF pore dimensions to enhance loading capacity and control the release of the loaded substance. Furthermore, MOFs can be modified before and after synthesis to improve their drug-loading capacity, structural stability, and cellular targeting (Lawson et al., 2021). Wu et al. created an active targeted medication delivery method to treat hypertrophic scars locally. Improved efficacy on hypertrophic scars was achieved using this drug delivery technology to regulate Wnt/β-catenin and JAK2/STAT pathways and downregulate collagens I and III expression. This system had superior performance compared to those without hypertrophic scar fibroblast (HSF) functionalization or the aid of microneedles (Wu et al., 2021). Pham et al. investigated the loading and release of a model drug, ibuprofen, by two iron-based MOFs *in vitro*. The results showed that the three-dimensional cage-like structure of MIL-88 had lower toxicity and better control of drug release than MIL-53 (Pham et al., 2020).

In bioimaging, MOFs are influenced by biomolecules that can be endowed with intrinsic fluorescent properties to serve as light sources for optical imaging. MOFs imbued with elements such as Au, Fe^3+^/Fe^2+^, Gd^3+^, Mn^2+^, fluorescent dyes, upconversion nanoparticles such as NaYF_4_, quantum dots, graphene, or nanotubes, can be utilized as an imaging tools (Liu et al., 2022). Liang et al. developed a method to integrate the near-infrared dye IR-3C and Ln^3+^ into MOFs to create strong near-infrared-II emissions. This bioimaging performance can delineate the vessels, spine, and lymph of mice and differentiate vessels with acute vascular inflammation *in vivo* (Liang et al., 2022). Li et al. fabricated a novel UiO-type MOF with high-order multiphoton excited fluorescence reactivity. The MOF has an extended π-electron system, improved charge transfer, enhanced dipole moment, and weakened π-π stacking interactions. Thus exhibits outstanding high-order multiphoton excited fluorescence performance in NIR-II light-induced fluorescence imaging (Li B. et al., 2022).

In nanoparticles, various nanoparticles can be integrated into MOF-based nanocomposites. These nanocomposites have potential in chemotherapy, photodynamic therapy, biocatalysts, starvation, and thermotherapy (Ge et al., 2022). Feng et al. developed a biodegradable oxygen-producing nanoplatform, Ini@PM-HP, made of poly (ADP-ribose) polymerase inhibitor PCN-224 and poly (dopamine-modified hyaluronic acid). This nanoplatform helped reduce the effects of oxygen deprivation in the tumor microenvironment on therapeutic efficacy (Feng et al., 2022). Zhou et al. produced a self-unpacking capsule that enables pH-triggered delivery of orally administered peptides or proteins by encapsulating amphiphilic hydrogel-coated MOF nanoparticles that prevent gastrointestinal degradation and facilitate penetration of the intestinal mucosa (Zhou et al., 2021).

In antibacterial applications, MOFs have desirable properties such as releasing antibacterial metal ions or organic linkers, accommodating and releasing large amounts of antimicrobial agents, generating reactive oxygen species, controlling/stimulating decomposition, strong interaction with bacterial membranes, and intense interaction with bacterial membranes (Li et al., 2021; Han et al., 2022). Chen et al. designed Zn-BTC, a nanoscale zinc-based MOF that can control the release of Zn^2+^. This material has an effective bactericidal impact on several drug-resistant bacteria, lowering Methicillin-resistant *Staphylococcus aureus* by 41.4% and *Escherichia coli* by 47.2% (Chen et al., 2022). Hu et al. combined ultrasmall gold nanoparticles and ultrathin 2D MOFs (UsAuNPs/MOFs). In addition to the benefits of both UsAuNPs and UsAuNPs/MOFs, they exhibit remarkable peroxidase-like activity toward breaking hydrogen peroxide into dangerous hydroxyl radicals. The UsAuNPs/MOF nanozyme demonstrates effective antibacterial activity against Gram-negative and Gram-positive microorganisms when combined with a low concentration of hydrogen peroxide (Hu W. C. et al., 2020).

### 4.3 Research frontiers and hotspots

Burst keywords represent emerging trends and research frontiers (Zhu and Zhang, 2021). As shown in Figure 8, we used the burst detection function of CiteSpace to identify burst keywords in these publications and found two current research hotspots in chemodynamic therapy (2020–2022) and hydrogen peroxide (2020–2022). We think these terms represent the future frontiers for biomedical applications of MOFs research.

Chemodynamic therapy is an *in-situ* treatment that generates hydroxyl radicals at tumor locations by the Fenton or Fenton-like reaction (Zhang et al., 2016; Tang et al., 2019). The Fenton reaction generates hydroxide and hydroxyl radicals via a reaction between Fe^2+^ and hydrogen peroxide (Gupta et al., 2016). In recent years, with the advancement of nanomedicine, the practical applications of chemodynamic therapy have been further developed due to its advantages, such as tumor specificity, the lack of necessary external stimulation, and fewer side effects (Jana and Zhao, 2022). Studies suggest that some functionalized MOFs can release metal ions within the tumor microenvironment. These metal ions can then act as catalysts for Fenton or Fenton-like reactions that produce hydroxyl radicals which can eradicate cancerous cells (Tan et al., 2022). Given this ability, functionalized MOFs are a promising material for chemodynamic therapy-based cancer therapy.

Deng et al. combined MOF modified by polydopamine and IR820 loaded with piperonasin to create a combination therapeutic nanosystem (MP@PI) combining chemodynamic and photothermal therapies designed to induce iron atrophy/thermal atrophy (Deng et al., 2022). Based on MOFs, Peng et al. created phosphate-responsive nanoparticles. These nanoparticles were loaded with glucose oxidases and doxorubicin, enabling them to display desirable synergistic therapeutic effects through enhanced Fenton reaction, starvation therapy, and combining chemotherapy (Peng et al., 2021).

Hydrogen peroxide is a reactive oxygen species that remains relatively stable in biological tissues and is involved in numerous physiological processes, including disease progression, intracellular signaling, cell physiology, and oxidative damage. Abnormal levels of hydrogen peroxide are associated with various oxidative stress disease states (Lismont et al., 2019), such as cancer (Meitzler et al., 2019), tumors (Yu Y. et al., 2021), asthma (Połomska et al., 2021), chronic obstructive pulmonary disease (Aggarwal et al., 2019), atherosclerosis (Harrison et al., 2003), diabetes (Wei et al., 2009), and neurodegenerative diseases (Cenini et al., 2019). MOF-based biosensors can measure the level of hydrogen peroxide in serum, plasma, supernatant from cell cultures, tissue/cell lysates, and exhaled breath condensates.

Using a coordination-assisted method, Mu et al. synthesized a novel iridium cluster-anchored MOF as a peroxidase-mimetic nanoreactor, called IrNCs@Ti-MOF, for the colorimetric detection of hydrogen peroxide-related biomarkers (Mu et al., 2022). Jiang et al. utilized graphene-like conductive MOF CuHHTP as a precursor to fabricate Cu2O nanoparticle@CuHTP heterojunction nanoarrays, which yielded satisfactory detection performance in measuring hydrogen peroxide concentration in urine and serum samples (Jiang et al., 2022).

### 4.4 Advantages and challenges in biomedical applications of MOFs

The characteristics of MOFs make them highly advantageous for applications in the biomedical field. MOFs can be synthesized with ease using various methods, including the hydrothermal method (Stock and Biswas, 2012), ultrasound-assisted method (Vaitsis et al., 2019), and mechanochemical method (Głowniak et al., 2021). Due to their large specific surface area and high loading rate, they are able to bind to small molecules and obtain biological activity (Mallakpour et al., 2022). The pore structure of MOFs can be readily adjusted to meet specific requirements by modifying the shape, length, and functional groups of linkers (Lu W. et al., 2014). MOFs can be endowed with specific functions through situ functionalization (Guo et al., 2022), pre-synthetic functionalization (López-Cabrelles et al., 2018), and post-synthetic modification (Younes et al., 2022). The metal ions and organic ligands released by MOFs can give them specific functions (Taheri et al., 2021). Additionally, MOFs can be designed to respond to external stimuli, such as temperature, pH, and light, to achieve controlled release of the drug (Wang et al., 2020).

Despite the many advantages of MOFs, their application in the biomedical field still faces some challenges. Biotoxicity is a challenge for MOFs. Metal ions and organic ligands released during decomposition, as well as organic solvents left in the pores during synthesis, may affect the biocompatibility and toxicity of MOFs. The toxic effects of MOFs still need to be further investigated in more *in vivo* and *in vitro* experiments (Kumar et al., 2019; Sajid and Ihsanullah, 2020). The stability of MOFs *in vivo* is also a challenge. MOFs may be affected by the biological environment, leading to structural changes or decomposition, which in turn affects their application effects (Singh et al., 2021).

## 5 Conclusion

Thus far, there has been a significant lack of bibliometric analysis regarding using MOFs in biomedical applications. Our study filled this void by performing a systematic bibliometric analysis of the literature on this topic by integrating both bibliometric tools and manual review.

The current research hotspots were identified by burst keyword analysis. Chemodynamic therapy and hydrogen peroxide are current research frontiers and hot spots. Functionalized MOFs can release metal ions into the tumor microenvironment and catalyze Fenton or Fenton-like reactions to produce hydroxyl radicals that kill tumor cells, making them promising materials for chemodynamic therapy. MOF-based biosensors measure hydrogen peroxide levels in serum, plasma, supernatants of cell cultures, tissue/cell lysates, and exhaled gas condensates for disease diagnosis. MOFs have a wide range of research prospects for biomedical applications.

The limitation of this study is that we could only analyze published articles, resulting in findings with a certain lag. For unpublished articles, ongoing research, articles not included in the Web of Science Core Collection, and non-English articles were not included in our study.

## Data Availability

The original contributions presented in the study are included in the article/supplementary material, further inquiries can be directed to the corresponding authors.

## References

[B1] Abánades Lázaro I. Forgan R. S. (2019). Application of zirconium MOFs in drug delivery and biomedicine. Coord. Chem. Rev. 380, 230–259. 10.1016/j.ccr.2018.09.009

[B2] Afzalinia A. Mirzaee M. (2020). Ultrasensitive fluorescent miRNA biosensor based on a “sandwich” oligonucleotide hybridization and fluorescence resonance energy transfer process using an Ln (III)-MOF and Ag nanoparticles for early cancer diagnosis: Application of central composite design. ACS Appl. Mater. interfaces 12, 16076–16087. 10.1021/acsami.0c00891 32207913

[B3] Aggarwal T. Wadhwa R. Thapliyal N. Sharma K. Rani V. Maurya P. K. (2019). Oxidative, inflammatory, genetic, and epigenetic biomarkers associated with chronic obstructive pulmonary disorder. J. Cell. physiology 234, 2067–2082. 10.1002/jcp.27181 30171697

[B4] Aria M. Cuccurullo C. (2017). bibliometrix: An R-tool for comprehensive science mapping analysis. J. Inf. 11, 959–975. 10.1016/j.joi.2017.08.007

[B5] Awasthi G. Shivgotra S. Nikhar S. Sundarrajan S. Ramakrishna S. Kumar P. (2022). Progressive trends on the biomedical applications of metal organic frameworks. Polymers 14, 4710. 10.3390/polym14214710 36365701 PMC9658682

[B6] Cenini G. Lloret A. Cascella R. (2019). Oxidative stress in neurodegenerative diseases: From a mitochondrial point of view. Oxidative Med. Cell. Longev. 2019, 1. 18. 10.1155/2019/2105607 PMC6532273 31210837

[B7] Chen C. Feng X. Zhu Q. Dong R. Yang R. Cheng Y. (2019). Microwave-assisted rapid synthesis of well-shaped MOF-74 (Ni) for CO2 efficient capture. Inorg. Chem. 58, 2717–2728. 10.1021/acs.inorgchem.8b03271 30720271

[B8] Chen C. Leydesdorff L. (2014). Patterns of connections and movements in dual‐map overlays: A new method of publication portfolio analysis. J. Assoc. Inf. Sci. Technol. 65, 334–351. 10.1002/asi.22968

[B9] Chen C. (2018). “Visualizing and exploring scientific literature with Citespace: An introduction,” in Proceedings of the 2018 Conference on Human Information Interaction & Retrieval, March 11 - 15, 2018, New Brunswick NJ USA, 369–370.

[B10] Chen Y. Cai J. Liu D. Liu S. Lei D. Zheng L. (2022). Zinc-based metal organic framework with antibacterial and anti-inflammatory properties for promoting wound healing. Regen. Biomater. 9, rbac019. 10.1093/rb/rbac019 35493287 PMC9046580

[B11] Cooper I. D. (2015). Bibliometrics basics. J. Med. Libr. Assoc. JMLA 103, 217–218. 10.3163/1536-5050.103.4.013 26512226 PMC4613387

[B12] Cunha D. Ben Yahia M. Hall S. Miller S. R. Chevreau H. Elkaïm E. (2013). Rationale of drug encapsulation and release from biocompatible porous metal–organic frameworks. Chem. Mater. 25, 2767–2776. 10.1021/cm400798p

[B13] Della Rocca J. Liu D. Lin W. (2011). Nanoscale metal–organic frameworks for biomedical imaging and drug delivery. Accounts Chem. Res. 44, 957–968. 10.1021/ar200028a PMC3777245 21648429

[B14] Deng H. Zhang J. Yang Y. Yang J. Wei Y. Ma S. (2022). Chemodynamic and photothermal combination therapy based on dual-modified metal-organic framework for inducing tumor ferroptosis/pyroptosis. ACS Appl. Mater Interfaces 14, 24089–24101. 10.1021/acsami.2c00574 35588091

[B15] Ding Q. Xu Z. Zhou L. Rao C. Li W. Muddassir M. (2022). A multimodal Metal-Organic framework based on unsaturated metal site for enhancing antitumor cytotoxicity through Chemo-Photodynamic therapy. J. colloid interface Sci. 621, 180–194. 10.1016/j.jcis.2022.04.078 35461133

[B16] Du C. Zhou M. Jia F. Ruan L. Lu H. Zhang J. (2021a). D-arginine-loaded metal-organic frameworks nanoparticles sensitize osteosarcoma to radiotherapy. Biomaterials 269, 120642. 10.1016/j.biomaterials.2020.120642 33440291

[B17] Du L. Chen W. Zhu P. Tian Y. Chen Y. Wu C. (2021b). Applications of functional metal‐organic frameworks in biosensors. Biotechnol. J. 16, 1900424. 10.1002/biot.201900424 32271998

[B18] Feng L. Chen M. Li R. Zhou L. Wang C. Ye P. (2022). Biodegradable oxygen-producing manganese-chelated metal organic frameworks for tumor-targeted synergistic chemo/photothermal/photodynamic therapy. Acta Biomater. 138, 463–477. 10.1016/j.actbio.2021.10.032 34718179

[B19] Furukawa H. Cordova K. E. O’keeffe M. Yaghi O. M. (2013). The chemistry and applications of metal-organic frameworks. Science 341, 1230444. 10.1126/science.1230444 23990564

[B20] Ge X. Wong R. Anisa A. Ma S. (2022). Recent development of metal-organic framework nanocomposites for biomedical applications. Biomaterials 281, 121322. 10.1016/j.biomaterials.2021.121322 34959029

[B21] Głowniak S. Szczęśniak B. Choma J. Jaroniec M. (2021). Mechanochemistry: Toward green synthesis of metal–organic frameworks. Mater. Today 46, 109–124. 10.1016/j.mattod.2021.01.008

[B22] Gu C. Wang Z. Pan Y. Zhu S. Gu Z. (2023). Tungsten‐based nanomaterials in the biomedical field: A bibliometric analysis of research progress and prospects. Adv. Mater. 35, 2204397. 10.1002/adma.202204397 35906814

[B23] Guo H. Liu L. Hu Q. Dou H. (2022). Monodisperse ZIF-8@dextran nanoparticles co-loaded with hydrophilic and hydrophobic functional cargos for combined near-infrared fluorescence imaging and photothermal therapy. Acta Biomater. 137, 290–304. 10.1016/j.actbio.2021.10.006 34637934

[B24] Gupta P. Lakes A. Dziubla T. (2016). “Chapter one - a free radical primer,” in Oxidative stress and biomaterials. Editors Dziubla T. Butterfield D. A. (Massachusetts, United States: Academic Press), 1–33.

[B25] Han D. Liu X. Wu S. (2022). Metal organic framework-based antibacterial agents and their underlying mechanisms. Chem. Soc. Rev. 51, 7138–7169. 10.1039/D2CS00460G 35866702

[B26] Harrison D. Griendling K. K. Landmesser U. Hornig B. Drexler H. (2003). Role of oxidative stress in atherosclerosis. Am. J. Cardiol. 91, 7–11. 10.1016/s0002-9149(02)03144-2 12645638

[B27] Heine M. H. (1998). Bradford ranking conventions and their application to a growing literature. J. Documentation 54, 303–331. 10.1108/EUM0000000007173

[B28] Hirsch J. E. (2007). Does the h index have predictive power? Proc. Natl. Acad. Sci. 104, 19193–19198. 10.1073/pnas.0707962104 18040045 PMC2148266

[B29] Horcajada P. Chalati T. Serre C. Gillet B. Sebrie C. Baati T. (2010). Porous metal–organic-framework nanoscale carriers as a potential platform for drug delivery and imaging. Nat. Mater. 9, 172–178. 10.1038/nmat2608 20010827

[B30] Horcajada P. Gref R. Baati T. Allan P. K. Maurin G. Couvreur P. (2012). Metal–organic frameworks in biomedicine. Chem. Rev. 112, 1232–1268. 10.1021/cr200256v 22168547

[B31] Hu C. Bai Y. Hou M. Wang Y. Wang L. Cao X. (2020a). Defect-induced activity enhancement of enzyme-encapsulated metal-organic frameworks revealed in microfluidic gradient mixing synthesis. Sci. Adv. 6, eaax5785. 10.1126/sciadv.aax5785 32064336 PMC6989138

[B32] Hu W. C. Younis M. R. Zhou Y. Wang C. Xia X. H. (2020b). *In situ* fabrication of ultrasmall gold nanoparticles/2D MOFs hybrid as nanozyme for antibacterial therapy. Small 16, 2000553. 10.1002/smll.202000553 32372554

[B33] Hu Z. Deibert B. J. Li J. (2014). Luminescent metal–organic frameworks for chemical sensing and explosive detection. Chem. Soc. Rev. 43, 5815–5840. 10.1039/c4cs00010b 24577142

[B34] Huang Y. Ren J. Qu X. (2019). Nanozymes: Classification, catalytic mechanisms, activity regulation, and applications. Chem. Rev. 119, 4357–4412. 10.1021/acs.chemrev.8b00672 30801188

[B35] James S. L. (2003). Metal-organic frameworks. Chem. Soc. Rev. 32, 276–288. 10.1039/b200393g 14518181

[B36] Jana D. Zhao Y. (2022). “Strategies for enhancing cancer chemodynamic therapy performance,” in Exploration (New Jersey, United States: Wiley Online Library), 20210238. 10.1002/EXP.20210238 PMC10191001 37323881

[B37] Jiang L. Wang H. Rao Z. Zhu J. Li G. Huang Q. (2022). *In situ* electrochemical reductive construction of metal oxide/metal-organic framework heterojunction nanoarrays for hydrogen peroxide sensing. J. Colloid Interface Sci. 622, 871–879. 10.1016/j.jcis.2022.04.095 35561607

[B38] Kitagawa S. (2014). Metal–organic frameworks (MOFs). Chem. Soc. Rev. 43, 5415–5418. 10.1039/C4CS90059F 25011480

[B39] Kıyıkçı H. F. Avcı O. Büyüksünetçi Y. T. Timur S. Anık Ü. (2023). Oxidase mimicking Co/2Fe MOF included biosensor for sialic acid detection. Talanta 254, 124166. 10.1016/j.talanta.2022.124166 36493566

[B40] Kreno L. E. Leong K. Farha O. K. Allendorf M. Van Duyne R. P. Hupp J. T. (2012). Metal–organic framework materials as chemical sensors. Chem. Rev. 112, 1105–1125. 10.1021/cr200324t 22070233

[B41] Kumar P. Anand B. Tsang Y. F. Kim K.-H. Khullar S. Wang B. (2019). Regeneration, degradation, and toxicity effect of MOFs: Opportunities and challenges. Environ. Res. 176, 108488. 10.1016/j.envres.2019.05.019 31295665

[B42] Lan G. Ni K. Veroneau S. S. Feng X. Nash G. T. Luo T. (2019). Titanium-based nanoscale metal–organic framework for type I photodynamic therapy. J. Am. Chem. Soc. 141, 4204–4208. 10.1021/jacs.8b13804 30779556

[B43] Lan G. Ni K. Xu Z. Veroneau S. S. Song Y. Lin W. (2018). Nanoscale metal–organic framework overcomes hypoxia for photodynamic therapy primed cancer immunotherapy. J. Am. Chem. Soc. 140, 5670–5673. 10.1021/jacs.8b01072 29665677 PMC6533908

[B44] Lawson H. D. Walton S. P. Chan C. (2021). Metal–organic frameworks for drug delivery: A design perspective. ACS Appl. Mater. interfaces 13, 7004–7020. 10.1021/acsami.1c01089 33554591 PMC11790311

[B45] Li B. Lu X. Tian Y. Li D. (2022a). Embedding multiphoton active units within metal–organic frameworks for turning on high‐order multiphoton excited fluorescence for bioimaging. Angew. Chem. Int. Ed. 61, e202206755. 10.1002/anie.202206755 35657165

[B46] Li B. Wen H. M. Cui Y. Zhou W. Qian G. Chen B. (2016). Emerging multifunctional metal–organic framework materials. Adv. Mater. 28, 8819–8860. 10.1002/adma.201601133 27454668

[B47] Li H. Li L. Lin R.-B. Zhou W. Zhang Z. Xiang S. (2019). Porous metal-organic frameworks for gas storage and separation: Status and challenges. EnergyChem 1, 100006. 10.1016/j.enchem.2019.100006 PMC11071076 38711814

[B48] Li M. Yin S. Lin M. Chen X. Pan Y. Peng Y. (2022b). Current status and prospects of metal-organic frameworks for bone therapy and bone repair. J. Mater. Chem. B 10, 5105–5128. 10.1039/d2tb00742h 35766423

[B49] Li R. Chen T. Pan X. (2021). Metal–organic-framework-based materials for antimicrobial applications. ACS Nano 15, 3808–3848. 10.1021/acsnano.0c09617 33629585

[B50] Li S.-Y. Cheng H. Xie B.-R. Qiu W.-X. Zeng J.-Y. Li C.-X. (2017). Cancer cell membrane camouflaged cascade bioreactor for cancer targeted starvation and photodynamic therapy. ACS Nano 11, 7006–7018. 10.1021/acsnano.7b02533 28665106

[B51] Lian X. Fang Y. Joseph E. Wang Q. Li J. Banerjee S. (2017). Enzyme–MOF (metal–organic framework) composites. Chem. Soc. Rev. 46, 3386–3401. 10.1039/c7cs00058h 28451673

[B52] Liang K. Ricco R. Doherty C. M. Styles M. J. Bell S. Kirby N. (2015). Biomimetic mineralization of metal-organic frameworks as protective coatings for biomacromolecules. Nat. Commun. 6, 7240. 10.1038/ncomms8240 26041070 PMC4468859

[B53] Liang T. Guo Z. He Y. Wang Y. Li C. Li Z. (2022). Cyanine‐doped lanthanide metal–organic frameworks for near‐infrared II bioimaging. Adv. Sci. 9, 2104561. 10.1002/advs.202104561 PMC8895151 35018733

[B54] Lismont C. Revenco I. Fransen M. (2019). Peroxisomal hydrogen peroxide metabolism and signaling in health and disease. Int. J. Mol. Sci. 20, 3673. 10.3390/ijms20153673 31357514 PMC6695606

[B55] Lismont M. Dreesen L. Wuttke S. (2017). Metal‐organic framework nanoparticles in photodynamic therapy: Current status and perspectives. Adv. Funct. Mater. 27, 1606314. 10.1002/adfm.201606314

[B56] Liu C. Wang J. Wan J. Yu C. (2021). MOF-on-MOF hybrids: Synthesis and applications. Coord. Chem. Rev. 432, 213743. 10.1016/j.ccr.2020.213743

[B57] Liu X. Yan Z. Zhang Y. Liu Z. Sun Y. Ren J. (2019). Two-dimensional metal–organic framework/enzyme hybrid nanocatalyst as a benign and self-activated cascade reagent for *in vivo* wound healing. Acs Nano 13, 5222–5230. 10.1021/acsnano.8b09501 31002497

[B58] Liu Y. Jiang T. Liu Z. (2022). Metal-organic frameworks for bioimaging: Strategies and challenges. Nanotheranostics 6, 143–160. 10.7150/ntno.63458 34976590 PMC8671950

[B59] López-Cabrelles J. Mañas-Valero S. Vitórica-Yrezábal I. J. Bereciartua P. Rodríguez-Velamazán J. Waerenborgh J. C. (2018). Isoreticular two-dimensional magnetic coordination polymers prepared through pre-synthetic ligand functionalization. Nat. Chem. 10, 1001–1007. 10.1038/s41557-018-0113-9 30150726

[B60] Lu K. Aung T. Guo N. Weichselbaum R. Lin W. (2018). Nanoscale metal–organic frameworks for therapeutic, imaging, and sensing applications. Adv. Mater. 30, 1707634. 10.1002/adma.201707634 PMC6586248 29971835

[B61] Lu K. He C. Lin W. (2014a). Nanoscale metal–organic framework for highly effective photodynamic therapy of resistant head and neck cancer. J. Am. Chem. Soc. 136, 16712–16715. 10.1021/ja508679h 25407895 PMC4277757

[B62] Lu W. Wei Z. Gu Z.-Y. Liu T.-F. Park J. Park J. (2014b). Tuning the structure and function of metal–organic frameworks via linker design. Chem. Soc. Rev. 43, 5561–5593. 10.1039/C4CS00003J 24604071

[B63] Mallakpour S. Nikkhoo E. Hussain C. M. (2022). Application of MOF materials as drug delivery systems for cancer therapy and dermal treatment. Coord. Chem. Rev. 451, 214262. 10.1016/j.ccr.2021.214262

[B64] Meitzler J. L. Konaté M. M. Doroshow J. H. (2019). Hydrogen peroxide-producing NADPH oxidases and the promotion of migratory phenotypes in cancer. Arch. Biochem. Biophys. 675, 108076. 10.1016/j.abb.2019.108076 31415727

[B65] Morris W. Briley W. E. Auyeung E. Cabezas M. D. Mirkin C. A. (2014). Nucleic acid–metal organic framework (MOF) nanoparticle conjugates. J. Am. Chem. Soc. 136, 7261–7264. 10.1021/ja503215w 24818877

[B66] Mu S. Deng Y. Xing Z. Rong X. He C. Cao S. (2022). Ir cluster-anchored MOFs as peroxidase-mimetic nanoreactors for diagnosing hydrogen peroxide-related biomarkers. ACS Appl. Mater Interfaces 14, 56635–56643. 10.1021/acsami.2c18676 36516976

[B67] Park J. Jiang Q. Feng D. Mao L. Zhou H.-C. (2016). Size-controlled synthesis of porphyrinic metal–organic framework and functionalization for targeted photodynamic therapy. J. Am. Chem. Soc. 138, 3518–3525. 10.1021/jacs.6b00007 26894555

[B68] Peng H. Qin Y. T. Feng Y. S. He X. W. Li W. Y. Zhang Y. K. (2021). Phosphate-degradable nanoparticles based on metal-organic frameworks for chemo-starvation-chemodynamic synergistic antitumor therapy. ACS Appl. Mater Interfaces 13, 37713–37723. 10.1021/acsami.1c10816 34340302

[B69] Pettinari C. Pettinari R. Di Nicola C. Tombesi A. Scuri S. Marchetti F. (2021). Antimicrobial MOFs. Coord. Chem. Rev. 446, 214121. 10.1016/j.ccr.2021.214121

[B70] Pham H. Ramos K. Sua A. Acuna J. Slowinska K. Nguyen T. (2020). Tuning crystal structures of iron-based metal–organic frameworks for drug delivery applications. ACS omega 5, 3418–3427. 10.1021/acsomega.9b03696 32118156 PMC7045591

[B71] Połomska J. Bar K. Sozańska B. (2021). Exhaled breath condensate—A non-invasive approach for diagnostic methods in asthma. J. Clin. Med. 10, 2697. 10.3390/jcm10122697 34207327 PMC8235112

[B72] Rodenas T. Luz I. Prieto G. Seoane B. Miro H. Corma A. (2015). Metal–organic framework nanosheets in polymer composite materials for gas separation. Nat. Mater. 14, 48–55. 10.1038/nmat4113 25362353 PMC4270742

[B73] Rosen A. S. Notestein J. M. Snurr R.Q. (2022). Realizing the data-driven, computational discovery of metal-organic framework catalysts. Curr. Opin. Chem. Eng. 35, 100760. 10.1016/j.coche.2021.100760

[B74] Sajid M. Ihsanullah R. (2020). “Chapter 17 - toxicity of nanoscale metal-organic frameworks in biological systems,” in Metal-organic frameworks for biomedical applications. Editor Mozafari M. (United Kingdom: Woodhead Publishing), 383–395.

[B75] Shao Y. Liu B. Di Z. Zhang G. Sun L.-D. Li L. (2020). Engineering of upconverted metal–organic frameworks for near-infrared light-triggered combinational photodynamic/chemo-/immunotherapy against hypoxic tumors. J. Am. Chem. Soc. 142, 3939–3946. 10.1021/jacs.9b12788 31968933

[B76] Simon-Yarza T. Mielcarek A. Couvreur P. Serre C. (2018). Nanoparticles of metal‐organic frameworks: On the road to *in vivo* efficacy in biomedicine. Adv. Mater. 30, 1707365. 10.1002/adma.201707365 29876985

[B77] Singh N. Qutub S. Khashab N. M. (2021). Biocompatibility and biodegradability of metal organic frameworks for biomedical applications. J. Mater. Chem. B 9, 5925–5934. 10.1039/d1tb01044a 34259304

[B78] Stock N. Biswas S. (2012). Synthesis of metal-organic frameworks (MOFs): Routes to various MOF topologies, morphologies, and composites. Chem. Rev. 112, 933–969. 10.1021/cr200304e 22098087

[B79] Taheri M. Ashok D. Sen T. Enge T. G. Verma N. K. Tricoli A. (2021). Stability of ZIF-8 nanopowders in bacterial culture media and its implication for antibacterial properties. Chem. Eng. J. 413, 127511. 10.1016/j.cej.2020.127511

[B80] Tan X. Liao D. Rao C. Zhou L. Tan Z. Pan Y. (2022). Recent advances in nano-architectonics of metal-organic frameworks for chemodynamic therapy. J. Solid State Chem. 10, 123352. 10.1016/j.jssc.2022.123352

[B81] Tang Z. Liu Y. He M. Bu W. (2019). Chemodynamic therapy: Tumour microenvironment‐mediated Fenton and fenton‐like reactions. Angew. Chem. 131, 946–956. 10.1002/anie.201805664 30048028

[B82] Tian H. Zhang M. Jin G. Jiang Y. Luan Y. (2021). Cu-MOF chemodynamic nanoplatform via modulating glutathione and H2O2 in tumor microenvironment for amplified cancer therapy. J. Colloid Interface Sci. 587, 358–366. 10.1016/j.jcis.2020.12.028 33360905

[B83] Vaitsis C. Sourkouni G. Argirusis C. (2019). Metal organic frameworks (MOFs) and ultrasound: A review. Ultrason. sonochemistry 52, 106–119. 10.1016/j.ultsonch.2018.11.004 30477790

[B84] Van Eck N. Waltman L. (2010). Software survey: VOSviewer, a computer program for bibliometric mapping. scientometrics 84, 523–538. 10.1007/s11192-009-0146-3 20585380 PMC2883932

[B85] Wang H.-S. (2017). Metal–organic frameworks for biosensing and bioimaging applications. Coord. Chem. Rev. 349, 139–155. 10.1016/j.ccr.2017.08.015

[B86] Wang X. Yang N. Li Q. He F. Yang Y. Wu B. (2019). Solvothermal synthesis of flower-string-like NiCo-MOF/MWCNT composites as a high-performance supercapacitor electrode material. J. Solid State Chem. 277, 575–586. 10.1016/j.jssc.2019.07.019

[B87] Wang Y. Yan J. Wen N. Xiong H. Cai S. He Q. (2020). Metal-organic frameworks for stimuli-responsive drug delivery. Biomaterials 230, 119619. 10.1016/j.biomaterials.2019.119619 31757529

[B88] Wei W. Liu Q. Tan Y. Liu L. Li X. Cai L. (2009). Oxidative stress, diabetes, and diabetic complications. Hemoglobin 33, 370–377. 10.3109/03630260903212175 19821780

[B89] Wu J. Wang X. Wang Q. Lou Z. Li S. Zhu Y. (2019). Nanomaterials with enzyme-like characteristics (nanozymes): Next-generation artificial enzymes (II). Chem. Soc. Rev. 48, 1004–1076. 10.1039/c8cs00457a 30534770

[B90] Wu M. X. Yang Y. W. (2017). Metal–organic framework (MOF)‐based drug/cargo delivery and cancer therapy. Adv. Mater. 29, 1606134. 10.1002/adma.201606134 28370555

[B91] Wu T. Hou X. Li J. Ruan H. Pei L. Guo T. (2021). Microneedle-Mediated biomimetic cyclodextrin metal organic frameworks for active targeting and treatment of hypertrophic scars. ACS Nano 15, 20087–20104. 10.1021/acsnano.1c07829 34792332

[B92] Xu D. Liu B. Wang J. Zhang Z. (2022). Bibliometric analysis of artificial intelligence for biotechnology and applied microbiology: Exploring research hotspots and frontiers. Front. Bioeng. Biotechnol. 10, 998298. 10.3389/fbioe.2022.998298 36277390 PMC9585160

[B93] Yan T. Zhang G. Yu K. Chai H. Tian M. Qu L. (2023). Smartphone light-driven zinc porphyrinic MOF nanosheets-based enzyme-free wearable photoelectrochemical sensor for continuous sweat vitamin C detection. Chem. Eng. J. 455, 140779. 10.1016/j.cej.2022.140779

[B94] Yang J. Dai D. Zhang X. Teng L. Ma L. Yang Y.-W. (2023). Multifunctional metal-organic framework (MOF)-based nanoplatforms for cancer therapy: From single to combination therapy. Theranostics 13, 295–323. 10.7150/thno.80687 36593957 PMC9800740

[B95] Yang J. Yang Y. W. (2020). Metal–organic frameworks for biomedical applications. Small 16, 1906846. 10.1002/smll.201906846 32026590

[B96] Ye Y. Zhao Y. Sun Y. Cao J. (2022). Recent progress of metal-organic framework-based photodynamic therapy for cancer treatment. Int. J. Nanomedicine 17, 2367–2395. 10.2147/IJN.S362759 35637838 PMC9144878

[B97] Younes H. A. Taha M. Mahmoud R. Mahmoud H. M. Abdelhameed R. M. (2022). High adsorption of sodium diclofenac on post-synthetic modified zirconium-based metal-organic frameworks: Experimental and theoretical studies. J. Colloid Interface Sci. 607, 334–346. 10.1016/j.jcis.2021.08.158 34509108

[B98] Yu J. Wei Z. Li Q. Wan F. Chao Z. Zhang X. (2021a). Advanced cancer starvation therapy by simultaneous deprivation of lactate and glucose using a MOF nanoplatform. Adv. Sci. (Weinh) 8, e2101467. 10.1002/advs.202101467 34363341 PMC8498878

[B99] Yu K. Lee Y.-R. Seo J. Y. Baek K.-Y. Chung Y.-M. Ahn W.-S. (2021b). Sonochemical synthesis of Zr-based porphyrinic MOF-525 and MOF-545: Enhancement in catalytic and adsorption properties. Microporous Mesoporous Mater. 316, 110985. 10.1016/j.micromeso.2021.110985

[B100] Yu K. Li M. Chai H. Liu Q. Hai X. Tian M. (2023). MOF-818 nanozyme-based colorimetric and electrochemical dual-mode smartphone sensing platform for *in situ* detection of H2O2 and H2S released from living cells. Chem. Eng. J. 451, 138321. 10.1016/j.cej.2022.138321

[B101] Yu Y. Peng J. Pan M. Ming Y. Li Y. Yuan L. (2021c). A nonenzymatic hydrogen peroxide electrochemical sensing and application in cancer diagnosis. Small Methods 5, e2001212. 10.1002/smtd.202001212 34928089

[B102] Yuan H. Li N. Fan W. Cai H. Zhao D. (2022). Metal‐organic framework based gas sensors. Adv. Sci. 9, 2104374. 10.1002/advs.202104374 PMC8867161 34939370

[B103] Zhang C. Bu W. Ni D. Zhang S. Li Q. Yao Z. (2016). Synthesis of iron nanometallic glasses and their application in cancer therapy by a localized Fenton reaction. Angew. Chem. Int. Ed. 55, 2101–2106. 10.1002/anie.201510031 26836344

[B104] Zhang G. Ma Y. Chai H. Yu K. Li Y. Wang S. (2022a). Porphyrinic metal–organic framework@ alumina nanocomposite fluorescent probe: Two-stage stimuli-responsive behavior and phosphate sensing. Sensors Actuators B Chem. 370, 132395. 10.1016/j.snb.2022.132395

[B105] Zhang G. Maddock J. Nenoff T. M. Denecke M. A. Yang S. Schröder M. (2022b). Adsorption of iodine in metal–organic framework materials. Chem. Soc. Rev. 51, 3243–3262. 10.1039/d0cs01192d 35363235 PMC9328120

[B106] Zhang J.-W. Zhang H.-T. Du Z.-Y. Wang X. Yu S.-H. Jiang H.-L. (2014). Water-stable metal–organic frameworks with intrinsic peroxidase-like catalytic activity as a colorimetric biosensing platform. Chem. Commun. 50, 1092–1094. 10.1039/c3cc48398c 24317416

[B107] Zhang Y. Wang F. Liu C. Wang Z. Kang L. Huang Y. (2018). Nanozyme decorated metal–organic frameworks for enhanced photodynamic therapy. ACS Nano 12, 651–661. 10.1021/acsnano.7b07746 29290107

[B108] Zheng H. Zhang Y. Liu L. Wan W. Guo P. NystroM A. M. (2016). One-pot synthesis of metal–organic frameworks with encapsulated target molecules and their applications for controlled drug delivery. J. Am. Chem. Soc. 138, 962–968. 10.1021/jacs.5b11720 26710234

[B109] Zhou H.-C. Long J. R. Yaghi O. M. (2012). Introduction to metal–organic frameworks. Washington, D.C.: ACS Publications. 10.1021/cr300014x 22280456

[B110] Zhou Y. Chen Z. Zhao D. Li D. He C. Chen X. (2021). A pH‐triggered self‐unpacking capsule containing zwitterionic hydrogel-coated MOF nanoparticles for efficient oral exendin‐4 delivery. Adv. Mater. 33, 2102044. 10.1002/adma.202102044 34216408

[B111] Zhu H. Zhang Z. (2021). Emerging trends and research foci in cataract genes: A bibliometric and visualized study. Front. Genet. 12, 610728. 10.3389/fgene.2021.610728 34434212 PMC8381374

[B112] Zhu S. Liu Y. Gu Z. Zhao Y. (2022). Research trends in biomedical applications of two-dimensional nanomaterials over the last decade–a bibliometric analysis. Adv. Drug Deliv. Rev. 188, 114420. 10.1016/j.addr.2022.114420 35835354

[B113] Zhuang J. Kuo C.-H. Chou L.-Y. Liu D.-Y. Weerapana E. Tsung C.-K. (2014). Optimized metal–organic-framework nanospheres for drug delivery: Evaluation of small-molecule encapsulation. ACS Nano 8, 2812–2819. 10.1021/nn406590q 24506773

[B114] Zhuang Z. Mai Z. Wang T. Liu D. (2020). Strategies for conversion between metal–organic frameworks and gels. Coord. Chem. Rev. 421, 213461. 10.1016/j.ccr.2020.213461

